# Synthesis and Bioactivity Investigation of Novel 2-({2-[(Dialkylamino) Methyl] Quinazolin-4-one-3-yl} Methyl) Benzonitrile Derivatives as Dipeptidyl Peptidase-4 Inhibitory Agent

**DOI:** 10.5812/ijpr-145406

**Published:** 2025-02-24

**Authors:** Arif Arrahman, Noer Luthfianeu Edsyah, Theresia Thiofani, Hanifah Sakinatun Khalidah, Laila Fauziah, Anjani Widyasintia, Benson Benson, Kevin Tanu Putra, Hayun Hayun

**Affiliations:** 1 Faculty of Pharmacy, Universitas Indonesia, Depok 16424, West Java, Indonesia

**Keywords:** Quinazolinone Derivatives, Quinazolin-4-one-3-yl-methylbenzonitrile, Synthesis, DPP-4 Inhibitor, Molecular Docking

## Abstract

**Background:**

Quinazolinone derivatives have been documented to exhibit antidiabetic properties via the mechanism of dipeptidyl peptidase-4 (DPP-4) inhibition.

**Objectives:**

To prepare and investigate the DPP-4 inhibitory activity in vitro and in silico of a series of novel 2-({2-[(dialkylamino)methyl]quinazolin-4-one-3-yl}methyl)benzonitrile derivatives.

**Methods:**

The compounds were synthesized, and the chemical structures were confirmed through spectroscopic techniques. The in vitro DPP-4 inhibitory activity was assessed using an assay kit. Additionally, an in silico study was conducted using molecular docking methods to analyze the occurring binding interactions.

**Results:**

The title compounds exhibited good inhibition against DPP-4 enzyme activity (IC_50_: 1.4621 to 6.7805 µM). Among the compounds studied, the compound having morpholino-methyl substituted at C-2 (5d) exhibited the highest potency in DPP-4 inhibitory activity. Their activities were lower than sitagliptin as the reference standard with IC_50_: 0.0236 µM and lead compound. In the in silico study, the compounds bound against the DPP-4 enzyme, with affinity values similar to those of sitagliptin. However, only compound 5f showed an interaction orientation and amino acid residues that were somewhat similar to those observed in the interaction between the DPP-4 enzyme and sitagliptin, as well as in the interaction between the DPP-4 enzyme and the lead compound.

**Conclusions:**

A series of novel 2-({2-[(dialkylamino)methyl]quinazolin-4-one-3-yl}methyl)benzonitrile derivatives have been synthesized successfully. All the synthesized compounds had lower DPP-4 inhibitory activity than sitagliptin and the lead compound. The lower bioactivity was predicted due to the differences in the interaction between the synthesized and lead compounds against the DPP-4 enzyme.

## 1. Background

**Figure 1.**
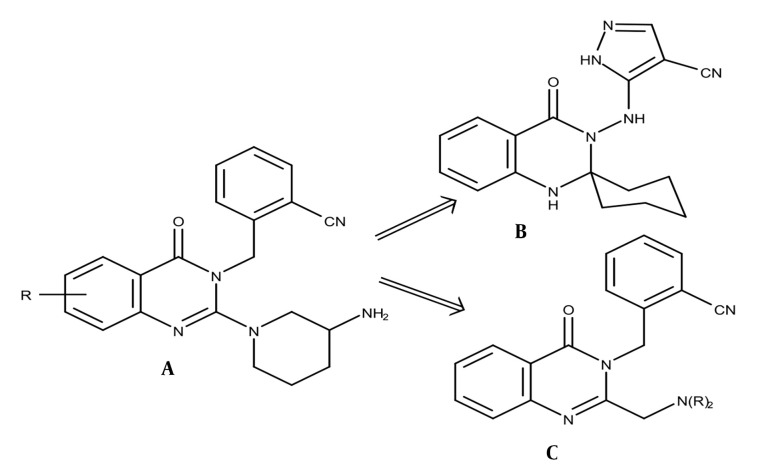
A and B, the DPP-4 inhibitor quinazolin-4-on derivatives; C, the title compound

Quinazolinone derivatives have been documented to possess a diverse range of bioactivities (1-8). Some compounds exhibit antidiabetic properties through dipeptidyl peptidase-4 (DPP-4) inhibition (5-8). A series of quinazolin-4-one compounds, substituted with methyl-benzonitrile at N-3 and 3-aminopiperidine at C-2 (Figure 1A), demonstrated potent and specific inhibition of DPP-4. Among these, the fluorinated derivative displayed the highest level of inhibition and metabolic stability in vivo. However, it also inhibited CYP450 3A4 and strongly blocked the hERG channel. Replacement of the quinazolin-4-one with a pyrimidine-2,4(1H,3H)-dione led to the development of alogliptin, which exhibited potent and more specific inhibition of DPP-4 activity without inhibiting CYP-450 enzymes or blocking the hERG channel (5).

Another study found that substituting methyl-benzonitrile at N-3 and 3-aminopiperidine at C-2 with amino-pyrazole-4-carbonitrile and spiro cyclohexane (Figure 1B) significantly enhanced the inhibitory potency of DPP-4. This modification resulted in a remarkable increase in activity, approximately 102 times stronger than linagliptin, which was used as a reference drug. Additionally, this modified compound exhibited a rapid onset of action and a prolonged duration of up to 24 hours (6). Conversely, quinazolin-4-one clubbed thiazoline derivatives and quinazolin-4-one-pyrimidine hybrid derivatives showed moderate to good DPP-4 inhibitory activity compared to linagliptin (7, 8). Given these results, we propose that substituted quinazolin-4-ones (Figure 1C) merit further investigation as potential new DPP-4 inhibitory agents.

## 2. Objectives

**Figure 2.**
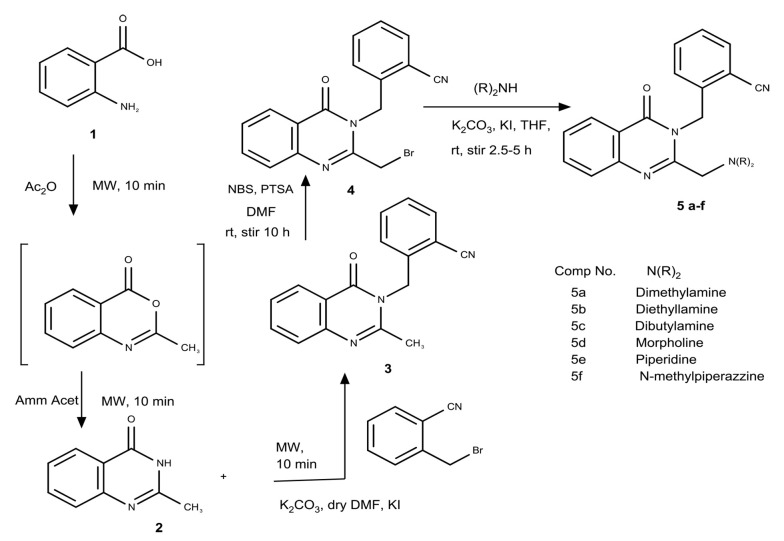
The preparation route of novel 2-({2-[(dialkylamino)methyl]quinazolin-4-one-3-yl} methyl)benzonitrile derivatives (5a-f); mw = microwave.

To discover novel quinazolin-4-one derivatives as DPP-4 inhibitory agents, we conducted the synthesis of a series of novel 2-({2-[(dialkylamino)methyl]quinazolin-4-one-3-yl}methyl)benzonitrile derivatives and investigated their potential bioactivities as DPP-4 inhibitory agents both in vitro and in silico (5a-f, Figure 2). 

## 3. Methods

### 3.1. Chemistry

#### 3.1.1. Analytical Methods

A Stuart scientific melting points apparatus was used to determine the compounds' melting points (uncorrected). Silica gel 60 (Merck, Germany) was utilized for the purification of compounds using a column chromatographic method. Thin layer chromatography (TLC) was employed to monitor the reaction progress and evaluate the product's purity. A Shimadzu 8400S FT-IR spectrophotometer, an Agilent NMR spectrometer, and a Waters High-Resolution Mass Spectrometer (H-MS) ESI R -TOP LCT premier XE were used to record the infrared spectra in KBr, ^1^H-NMR (500 MHz) and ^13^C-NMR (125 MHz) spectra in CDCl_3_, and mass spectra of the compounds, respectively.

#### 3.1.2. Preparation of 2-Methylquinazolin-4-one-3-yl (2)

Compound 2 was prepared using a microwave irradiation procedure following the reported methods (9-11).

#### 3.1.3. Preparation of 2-[(2-Methylquinazolin-4-one-3-yl)methyl]benzonitrile (3)

Compound 3 was prepared using a documented methodology with slight modifications (12). A solution of Compound 2 (0.096 mol) in anhydrous dimethylformamide (DMF) (157.8 mL) was prepared, and potassium carbonate (K_2_CO_3_) (0.1143 mol) was added. The mixture was irradiated in a microwave operating at 600 W/100°C for 3 minutes. Potassium iodide (KI) (0.036 mol) was then introduced and mixed for 15 minutes. Subsequently, a solution containing 2-(bromomethyl)benzonitrile (0.095 mol) in 50 mL of anhydrous DMF was gradually added to the mixture and agitated until a uniform composition was achieved. The mixture was exposed to microwave irradiation at 600 W/100°C for 10 minutes (monitored by TLC), allowed to cool, and then transferred into crushed ice while continuously stirred and supplemented with additional water to generate a suspension. The suspension was filtered, and the obtained solid was dried and recrystallized from ethanol, resulting in a yellow powder with a yield of 34%. IR (υ/cm^−1^): 3000 - 3100, 2950, 2220, 1660, 1550 - 1600, 1450; ^1^H-NMR (δ_ppm_, J (Hz)): 8.30 (dd, J = 8.1, 1H, H-5 quinazolinone), 7.78 (dt, J = 7.6, 1H, H-7 quinazolinone), 7.73 (dd, J = 7.7, 1H, H-8 quinazolinone), 7.66 (dd, J = 8.3, 1H, benzonitrile), 7.50 (m, overlap, 1H, H-6 quinazolinone and 1H, benzonitrile), 7.40 (dt, J = 7.6, 1H, benzonitrile), 7.07 (d, J = 7.9, 1H, benzonitrile), 5.61 (2H, s, Ar-CH_2_-N), 2.53 (3H, s, (N=C)CH_3_); ^13^C-NMR (δ_ppm_): 162.4 (C=O), 154.1 (C=N(N)), 147.4 (C_9_), 134.9 (C_7_), 127.1 (C_6_), 127.0 (C_8_), 126.4 (C_5_), 120.2 (C_10_), 139.6, 133.8, 133.3, 128.4, 127.3, and 111.2 (6C-Ar), 117.0 (C≡N), 45.5 (Ar-CH_2_-N-3), 23.4 (CH_3_).

#### 3.1.4. Preparation of 2-[(2-Bromomethylquinazolin-4-one-3-yl)methyl]benzonitrile (4)

Compound 4 was prepared using the reported methodology (11, 13, 14) with certain modifications. A solution of compound 3 (23.75 mmol) in anhydrous DMF (95 mL) was mixed with N-bromosuccinimide (NBS) (23.75 mmol) and p-toluene sulfonic acid (2.375 mmol) and stirred at room temperature for 10 hours, monitored by TLC, until a by-product spot was identified. Subsequently, the mixture was transferred into crushed ice, and the precipitate was filtered and washed with distilled water, followed by drying in a vacuum oven at 70°C for one hour. Purification using column chromatography resulted in a pale-yellow crystal product with a yield of 38%. IR (υ/cm^−1^): 3000 - 3100, 2950, 2220, 1660, 1550 - 1600, 1450, 1225, 570; ^1^H-NMR (δ_ppm_, J (Hz)): 8.30 (td, J = 4.0, 1H, H-5 quinazolinone), 7.83 (dt, J = 7.5, 1H, H-7 quinazolinone), 7.74 (overlap, J = 8, 1H, H-8 quinazolinone and 1H, benzonitrile), 7.56 (dt, J = 7.5, 1H, H-6 quinazolinone), 7.52 (dt, J = 7.7, 1H, benzonitrile), 7.41 (dt, J = 7.5, 1H, benzonitrile), 7.04 (d, J = 7.9, 1H, benzonitrile), 5.76 (s, 2H, N-CH_2_-Ar), 4.33 (s, 2H, CH_2_-Br); ^13^C-NMR (δ_ppm_): 162.2 (C=O), 151.7 (C=N), 146.9 (C_9_), 135.2 (C_7_), 127.9 (C_6_), 127.4 (C_8_), 126.3 (C_5_), 120.8 (C_10_), 139.5, 133.7, 133.5, 128.5, 128.3, and 111.1 (6C-Ar), 117.0 (C≡N), 45.4 (N-CH_2_-Ar), 29.5 (CH_2_-Br).

#### 3.1.5. Preparation of 2-({2-[(Dialkylamino)methyl]quinazolin-4-one-3-yl}methyl)benzonitrile (5a-f)

Compounds 5a-f were prepared by modifying the N-alkylation of amino adamantane technique, as described in the synthesis of vildagliptin and its impurities (15, 16). A solution containing 1.5 mmol of the appropriate secondary amines in 5 mL tetrahydrofuran (THF) was mixed with K_2_CO_3_ (3.4 mmol) and KI (0.002 mmol). After cooling in an ice bath to 0°C, compound 4 (0.85 mmol) was introduced, agitated, and kept at 0°C for 1 hour, then allowed to warm to room temperature while being continuously stirred for 2.5 - 5 hours (monitored by TLC). The insoluble substance was separated by filtration, and the filtrate was then placed upon crushed ice. The precipitate was purified through filtration, washed with cold water, and recrystallized using an appropriate solvent, yielding pure 5a-f.

##### 3.1.5.1. 2-({2-[Dimethylaminomethyl]quinazolin-4-one-3-yl}methyl)benzonitrile (5a)

Yield: 62.24%, white powder, m.p. 146 - 148°C; IR (υ/cm^−1^): 3000 - 3100, 2800 - 2970, 2220, 1660, 1570 - 1600, 1450 - 1470, 1200; ^1^H-NMR (δ_ppm_, J (Hz)): 8.32 (d, J = 7.9, 1H, H-5 quinazolinone), 7.80 (t, J = 7.4, 1H, H-7 quinazolinone), 7.74 (d, J = 7.5, 1H, H-8 quinazolinone), 7.71 (d, J = 7.6, 1H, benzonitrile), 7.53 (t, J = 7.5, 1H, H-6 quinazolinone), 7.46 (t, J = 7.8, 1H, benzonitrile), 7.35 (t, J = 7.6, 1H, benzonitrile), 6.92 (d, J = 8, 1H, benzonitrile), 5.97 (s, 2H, N-CH_2_-Ar), 3.41 (s, 2H, (N=C)-CH_2_-N), 2.26 (s, 6H, N(CH_3_)2); ^13^C-NMR (δ_ppm_): 162.6 (C=O), 153.6 (C=N), 147.0 (C_9_), 140.8, 134.8 (C_7_), 127.6 (C_6_), 127.2 (C_8_), 125.6 (C_5_), 120.7 (C_10_), 133.4, 133.3, 127.7, 127.5, 110.8 (6C-Ar), 117.2 (C≡N), 64.1 ((N=C)CH_2_-N), 45.3 (N(CH_3_)2), 44.8 (N-CH_2_-Ar); ESI-MS (m/z): [M+H]_+_ found 319.1554, M = 318.1476; Exact MW for C_19_H_18_N_4_O = 318.1481, error 1.6 ppm.

##### 3.1.5.2. 2-({2-[Diethylaminomethyl]quinazolin-4-one-3-yl}methyl)benzonitrile (5b)

Yield: 74.99%, white powder, m.p. 114 - 116°C; IR (υ/cm^−1^): 3000 - 3100, 2800 - 2970, 2220, 1670, 1600, 1470, 1200; ^1^H-NMR (δ_ppm_, J (Hz)): 8.31 (d, J = 7.9, 1H, H-5 quinazolinone), 7.80 (t, J = 8.2, 1H, H-7 quinazolinone), 7.74 (d, J = 8.1, 1H, H-8 quinazolinone), 7.71 (d, J = 7.6, 1H, benzonitrile), 7.53 (t, J = 7.6, 1H, H-6 quinazolinone), 7.45 (t, J = 7.7, 1H, benzonitrile), 7.34 (t, J = 7.6, 1H, benzonitrile), 6.83 (d, J = 7.95, 1H, benzonitrile), 6.02 (s, 2H, N-CH_2_-Ar), 3.58 (s, 2H, (N=C)-CH_2_-N), 2.56 (q, 4H, N-(CH_2_)2-C), 0.93 (t, 6H, 2CH_3_-C); ^13^C-NMR (δ_ppm_): 162.5 (C=O), 154.4 (C=N), 147.2 (C_9_), 134.8 (C_7_), 127.4 (C_6_), 127.2 (C_8_), 125.1 (C_5_), 120.7 (C_10_), 140.8, 133.4, 133.3, 127.6, 127.5, and 110.7 (6C-Ar), 117.2 (C≡N), 59.4 ((N=C)CH_2_-N), 46.6 (N(CH_2_)2), 44.9 (N-CH_2_-Ar), 10.9 (2CH_3_-C); ESI-MS (m/z): [M+H]_+_ found 347.1872, M = 346.14794; Exact MW for C_21_H_22_N_4_O = 346.1794, error 0.0 ppm.

##### 3.1.5.3. 2-({2-[Dibutylaminomethyl]quinazolin-4-one-3-yl}methyl)benzonitrile (5c)

Yield: 79.75%, white crystal, m.p. 112 - 114°C; IR (υ/cm^−1^): 3000 - 3100, 2800 - 2970, 2220, 1670, 1600, 1470, 1200; ^1^H-NMR (δ_ppm_, J (Hz)): 8.30 (d, J = 7.9, 1H, H-5 quinazolinone), 7.80 (t, J = 8.0, 1H, H-7 quinazolinone), 7.76 (d, J = 7.9, 1H, H-8 quinazolinone), 7.71 (d, J = 7.5, benzonitrile), 7.53 (t, J = 7.1, 1H, H-6 quinazolinone), 7.45 (t, J = 7.3, 1H, benzonitrile), 7.34 (t, J = 7.5, 1H, benzonitrile), 6.81 (d, J = 7.9, 1H, benzonitrile), 6.05 (s, 2H, N-CH_2_-Ar), 3.58 (s, 2H, (N=C)-CH_2_-N), 2.48 (t, 4H, 2(N-CH_2_-C)), 1.33 (m, 4H, 2(C-CH_2_-C)), 1.23 (m, 4H, 2(C-CH_2_-C)), 0.82 (t, 6H, 2(C-CH_3_)); ^13^C-NMR (δ_ppm_): 162.4 (C=O), 154.4 (C=N), 147.2 (C_9_), 134.8 (C_7_), 127.4 (C_6_), 127.2 (C_8_), 124.9 (C_5_), 120.6 (C_10_), 140.8, 133.4, 127.6, 127.5, 110.8 (6C-Ar), 117.0 (C≡N), 60.5 ((N=C)CH_2_-N), 53.5 (N(CH_2_-)2), 44.7 (N-CH_2_-Ar), 28.3 (2(-CH_2_-)), 20.7 (2(-CH_2_-)), 14.1 (2CH_3_-); ESI-MS (m/z): [M+H]^+^ found 403.24932, M = 402.2415; Exact MW for C_25_H_30_N_4_O = 402.2420, error 1.2 ppm.

##### 3.1.5.4. 2-({2-[Morpholinomethyl]quinazolin-4-one-3-yl}methyl)benzonitrile (5d)

Yield: 51.12%, white powder, m.p. 128 - 130°C; IR (υ/cm^−1^): 3000 - 3100, 2800 - 2970, 2220, 1680, 1600, 1450, 1200, 1070; ^1^H-NMR (δ_ppm_, J (Hz)): 8.34 (d, J = 7.9, 1H, H-5 quinazolinone), 7.80 (t, J = 8.3, 1H, H-7 quinazolinone), 7.73 (t, J = 7.5, 1H, H-8 quinazolinone and 1H, benzonitrile), 7.55 (t, J = 7.9, 1H, H-6 quinazolinone), 7.48 (t, J = 7.2, 1H, benzonitrile), 7.37 (t, J = 7.5, 1H, benzonitrile), 6.93 (d, J = 7.9, 1H, benzonitrile), 5.92 (s, 2H, N-CH_2_-Ar), 3.52 (s, 2H, (N=C)CH_2_-N), 3.48 (t, 4H, O(CH_2_-)2 morpholine), 2.49 (t, 4H, N(CH_2_-)2 morpholine); ^13^C-NMR (δ_ppm_): 162.7 (C=O), 152.5 (C=N), 146.9 (C_9_), 134.9 (C_7_), 127.6 (C_6_), 127.3 (C_8_), 125.7 (C_5_), 120.7 (C_10_), 140.9, 133.5, 133.2, 127.8, 127.7, 110.7 (6C-Ar), 117.3 (C≡N), 66.6 ((N=C)CH_2_-N), 63.6 (N-CH_2_-Ar), 53.5 (O(CH_2_-)2 morpholine), 45.2 (N(CH_2_-)2 morpholine); ESI-MS (m/z): [M+H]_+_ found 361.1660, M = 360.1582; Exact MW for C_21_H_20_N_4_O_2_ = 360.1586, error 1.1 ppm.

##### 3.1.5.5. 2-({2-[Pyrrolidinomethyl]quinazolin-4-one-3-yl}methyl)benzonitrile (5e)

Yield: 50.09%, white-yellowish powder, m.p. 182 - 184 °C; IR (υ/cm^−1^): 3000 - 3100, 2800 - 2970, 2220, 1680, 1600, 1450, 1150; ^1^H-NMR (δ_ppm_, J (Hz)): 8.34 (dd, J = 7.9, 1H, H-5 quinazolinone), 7.79 (dt, J = 7.6, 1H, H-7 quinazolinone), 7.74 (dd, J = 8.1, 1H, H-8 quinazolinone), 7.70 (dd, J = 7.8, 1H, benzonitrile), 7.53 (dt, J = 7.5, 1H, H-6 quinazolinone), 7.45 (dt, J = 7.8, 1H, benzonitrile), 7.34 (dt, J = 7.6, 1H, benzonitrile), 6.94 (d, J = 7.9, 1H, benzonitrile), 5.92 (s, 2H, N-CH_2_-Ar), 3.64 (s, 2H, (N=C)CH_2_-N), 2.52 (m, 4H, N(CH_2_-)2 pyrrolidine), 1.62 (m, 4H, (CH_2_-)2 pyrrolidine).

^13^C-NMR (δ_ppm_): 162.7 (C=O), 154.2 (C=N), 147.2 (C_9_), 134.8 (C_7_), 127.4 (C_6_), 127.2 (C_8_), 125.6 (C_5_), 120.7 (C_10_), 141.1, 133.2, 133.1, 127.6, 127.5, 110.7 (6C-Ar), 117.3 (C≡N), 60.6 ((N=C)CH_2_-N), 53.8 (N-CH_2_-Ar), 45.1 (N(CH_2_-)2 pyrrolidine), 23.6 ((CH_2_-)2 pyrrolidine); ESI-MS (m/z): [M+H]^+^ found 345.1713, M = 344.1635; Exact MW for C_21_H_20_N_4_O_2_ = 344.1637, error 0.6 ppm.

##### 3.1.5.6. 2-({2-[N-methylpiperazinomethyl]quinazolin-4-one-3-yl}methyl)benzonitrile (5f)

Yield: 20.793%, white yellowish powder, m.p. 196 - 200°C; IR (υ/cm^−1^): 3000 - 3100, 2800 - 2970, 2220, 1680, 1600, 1450, 1120; ^1^H-NMR (δ_ppm_, J (Hz)): 8.34 (dd, J = 8.0, 1H, H-5 quinazolinone), 7.80 (dt, J = 6.9, 1H, H-7 quinazolinone), 7.72 (t, J = 7.7, 1H, H-8 quinazolinone and 1H, benzonitrile), 7.53 (dt, J = 7.6, 1H, H-6 quinazolinone), 7.47 (dt, J = 7.6, 1H, benzonitrile), 7.36 (t, J = 7.6, 1H, benzonitrile), 6.92 (d, J = 7.9, 1H, benzonitrile), 5.92 (s, 2H, N-CH_2_-Ar), 3.51 (s, 2H, (N=C)CH_2_-N), 2.54 (m, 8H, 2(N(CH_2_)2) piperazine), 2.21 (s, 3H, N-CH_3_); ^13^C-NMR (δ_ppm_): 162.7 (C=O), 152.8 (C=N), 146.9 (C_9_), 134.9 (C_7_), 127.6 (C_6_), 127.2 (C_8_), 125.7 (C_5_), 120.7 (C_10_), 140.9, 133.5, 133.2, 127.7, 110.7 (6C-Ar), 117.2 (C≡N), 63.1 ((N=C)CH_2_-N), 54.3 (N-(CH_2_-)2) piperazine, 52.6 (N-(CH_2_-)2) piperazine, 45.5 (N-CH_2_-Ar), 45.0 (N-CH_3_); ESI-MS (m/z): [M+H]_+_ found 374.1973, M = 373.1895; Exact MW for C_22_H_23_N_5_O = 373.1903, error -2.1 ppm.

### 3.2. Dipeptidyl Peptidase-4 Inhibitory Activity Assay

The in vitro DPP-4 inhibitory activity of the prepared compounds was evaluated using the DPP-4 inhibitor screening kit (MAK203) according to the test protocol with slight modifications (17). Briefly, mixtures of synthesized compound solutions (25 μL) at various final concentrations (0.025, 6.25, 12.5, and 25 μM) and DPP-4 solution (50 μL) in microwell plates were incubated at 37°C for 10 minutes. Substrate solutions (25 μL) were added to each well, covered, and incubated at 37°C for 30 minutes. Finally, fluorescence intensity was measured at λ_ex_ = 365 nm and λ_em_ = 415 - 445 nm using a microplate reader. Sitagliptin solution (20, 40, 60, and 80 nM final concentrations) was used as a positive control/reference. The following formula was used to calculate the percent inhibition (Equation 1):


$$Inhibition\;\left(\%\right)=\;\left[\frac{\left(initial\;activity-inhibitor\;activity\right)}{initial\;activity}\right]\times 100\;\%$$

The IC_50_ value was calculated from y = a + bx (linear equation) obtained from the relation of the inhibition activity (%) (y) and the concentration (x) of the substance being measured.

### 3.3. In Silico Study

#### 3.3.1. Preparation of Protein and Ligands

The crystal structure of the DPP-4 protein in complex with sitagliptin as a native ligand, with a resolution of 2.1 Å (PDB ID: 1X70; http://www.rcsb.org/) (18), was selected as the protein target in this study. The ligand and water molecules were separated from the protein using Python Molecular Viewer (PMV-1.5.7). Gasteiger charges and hydrogen atoms were added to the protein, while polar hydrogens and Kollman charges were assigned to each ligand. Finally, all the prepared ligands and the protein were converted into the PDBQT format (.pdbqt) using MGL tools and the Open Babel program (19, 20).

#### 3.3.2. Molecular Docking Simulation

Docking simulations for the test compounds (ligands) (5a-f) were performed using AutoDock Vina. The native and test ligands were simulated in different conformations to achieve the best binding interaction with the DPP-4 enzyme's binding site. The criteria were the lowest free energy of affinity (ΔG), calculated using the Lamarckian genetic algorithm (LGA). The LGA parameters refer to previous studies (21). The grid box used for molecular docking analysis consisted of 52 x 28 x 26 Å, with points spaced 0.375 Å apart, centered on the active site (XYZ coordinates: X = 40.926 Å; Y = 50.522 Å; Z = 35.031 Å). The results were visualized using PyMOL. The docking protocol's validity was proven by re-docking the native ligand into its original location 50 times and determining the root mean square deviation (RMSD) value.

## 4. Results and Discussion

The 2-({2-[(dialkylamino)methyl]quinazolin-4-one-3-yl}methyl)benzonitrile derivatives (5a-f) were prepared by synthesizing them stepwise, as shown in Figure 2. Anthranilamide (1) was reacted with acetic anhydride in a microwave at a power level of 30% for 10 minutes, followed by the addition of ammonium acetate, and heated in the microwave at a power level of 30% for another 10 minutes to provide 2-methyl-3,4-dihydroquinazolin-4-one (2). Compound 2 was then treated with 2-bromomethyl-benzonitrile with K_2_CO_3_ and KI as catalysts in anhydrous DMF in the microwave at a power level of 70% for 10 minutes to afford 2-[(2-methylquinazolin-4-one-3-yl)methyl]benzonitrile (3). Subsequently, compound 3 was brominated with NBS in anhydrous DMF with p-toluene sulfonic acid as a catalyst and stirred at room temperature for 10 hours to afford 2-[(2-bromomethylquinazolin-4-one-3-yl)methyl]benzonitrile (4). Finally, compound 4 was treated with corresponding secondary amines in THF with K_2_CO_3_ and KI as catalysts and mixed at room temperature for 2.5 - 5 hours to afford the target compounds (5a-f).

The IR spectra of 5a-f exhibited peaks in the region at 1660 - 1680 and 2220 cm^-1^, indicating the existence of carbonyl and carbonitrile groups, respectively. In the ^1^H-NMR, the proton peaks of methylene (CH_2_) connecting N-3 quinazolinone with benzonitrile and C-2 quinazolinone with the alkylamine were observed as singlet proton peaks in the region δ 5.92 - 6.05 and 3.41 - 3.64 ppm. The observation of carbon peaks in the region at δ 162.4 - 162.7 and 117.0 - 117.3 ppm of the ^13^C-NMR spectra confirmed the existence of carbonyl and carbonitrile groups (22). All the compounds’ MS spectra displayed the m/z values of their molecular ion peaks corresponding to their molecular formula.

**Figure 3.**
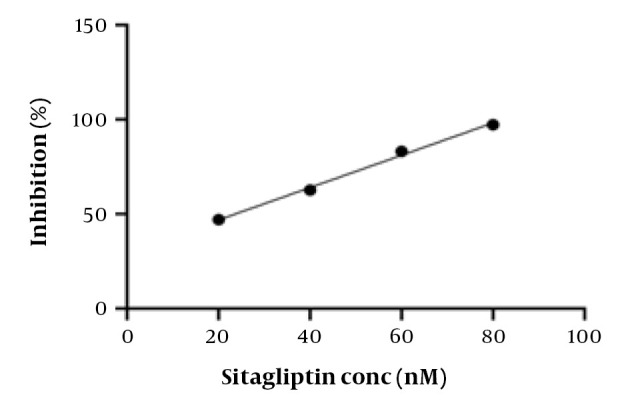
The inhibition (%) of dipeptidyl peptidase-4 (DPP-4) activity versus concentration (nM) curve of sitagliptin

**Figure 4.**
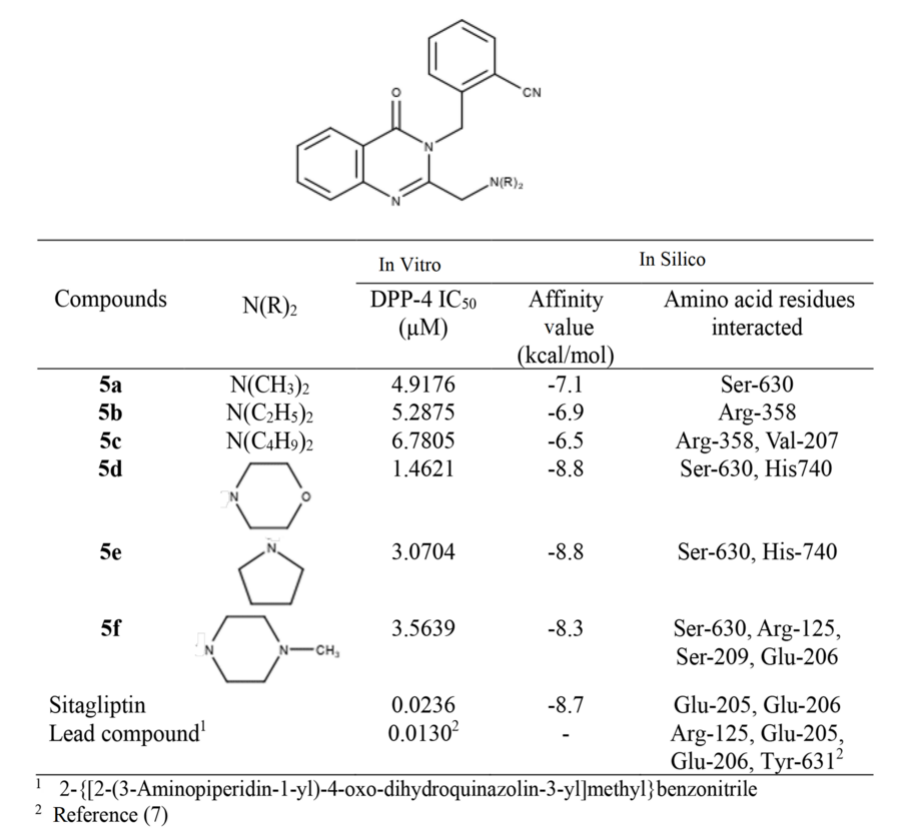
The IC_50_ value of in vitro inhibitory dipeptidyl peptidase-4 (DPP-4) activity and in silico affinity value of novel 2-({2-[(dialkylamino)methyl]quinazolin-4-one-3-yl}methyl)benzonitrile derivatives (7)

The investigation of the DPP-4 inhibitory activity of the prepared compounds was performed using a DPP-4 activity assay kit. The inhibition (%) versus concentration (nM) curve of sitagliptin (positive control or reference standard) is presented in Figure 3. The obtained IC_50_ value of sitagliptin was 23.6 nM. The difference from previously reported values (4, 23, 24) is possibly due to experimental condition differences. The title compounds exhibited good DPP-4 inhibitory activity (IC_50_: 1.4621 - 6.7805 µM) (Figure 4). Compound 5d, with a morpholino-methyl substitution at C-2, showed the highest activity. The bioactivity of the compounds was lower than that of sitagliptin and the lead compound (IC_50_: 13 nM) (6).

**Figure 5.**
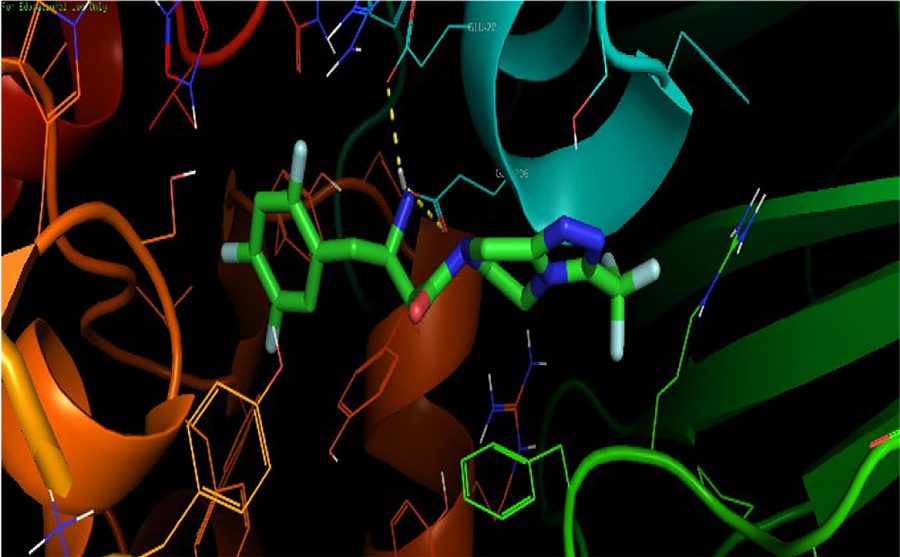
Binding interaction of sitagliptin in the dipeptidyl peptidase-4 (DPP-4)’s active site

In the in silico study, re-docking the native ligand resulted in a RMSD of 0.65 Å, which is less than 2 Å, indicating the validity of the docking process (21). The docking analysis of sitagliptin with the DPP-4 enzyme indicated an affinity value of -8.7 kcal/mol. The 2,4,5-trifluorophenyl moiety completely occupied the S1 hydrophobic pocket of the DPP-4 enzyme. Additionally, the (R)-β-amino group formed hydrogen bonds with Glu-205 and Glu-206 (Figure 5). These findings are consistent with previously reported results (25-27).

The synthesized compounds bound to the DPP-4 enzyme, exhibiting affinity values ranging from -6.5 to -8.8 kcal/mol. However, the amino acid residues involved in these interactions differed from those that interacted with sitagliptin and the lead compound, 2-{[2-(3-aminopiperidin-1-yl)quinazolin-4-one-3-yl]methyl}benzonitrile (Figure 4). Only compound 5f showed a somewhat similar interaction orientation to those observed in the interaction between the DPP-4 enzyme and the lead compound. The methyl-benzonitrile at N-3 filled the S1 pocket with the nitrile group, forming two hydrogen bonds with Arg-125 and Ser-630. At the same time, N-methyl-piperazine groups attached at C-2 established hydrogen bonds with Glu-205.

**Figure 6.**
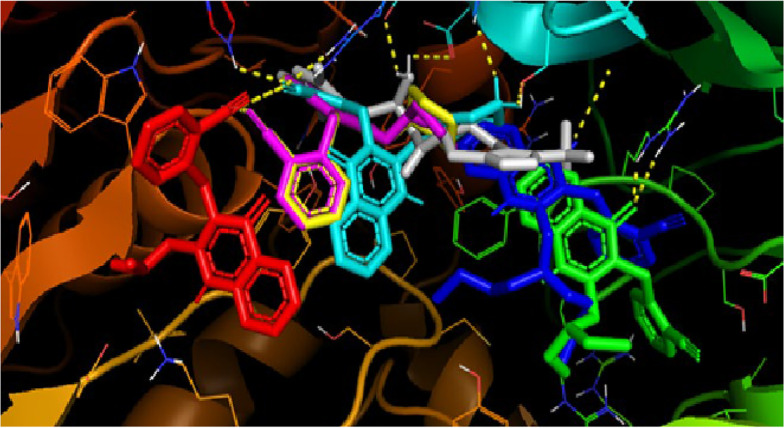
Comparison of the binding interaction between compounds 5a (red), 5b (green), 5c (blue), 5d (yellow), 5e (violet), 5f (light blue), and sitagliptin (white) in the dipeptidyl peptidase-4 (DPP-4)’s active site

Compounds 5a, 5b, and 5c did not occupy the S1 pocket, while compounds 5d and 5e occupied the S1 pocket but with different orientations. It was not the methyl-benzonitrile group that filled the S1 pocket; it was the quinazolin-4-one ring (Figure 6). In the interaction of the lead compound with the active site of the DPP-4 enzyme, the methyl-benzonitrile attached at N-3 effectively filled the S1 pocket with the nitrile group, forming a hydrogen bond with Arg-125. Meanwhile, the 3-aminopiperidine moieties attached at C-2 provided ionic interactions with Glu-205 and Glu-206, and the carbonyl at C-4 provided a hydrogen bond to Tyr-631 (5).

The differences in binding interactions between the synthesized and lead compounds to the DPP-4 enzyme likely contributed to the low bioactivity of the synthesized compounds. The study's results could be valuable for designing new molecules in the investigation of DPP-4 inhibitor agents with excellent and selective activity.

## 5. Conclusions

A series of novel derivatives of 2-({2-[(dialkylamino)methyl]quinazolin-4-one-3-yl}methyl)benzonitrile was successfully synthesized and investigated for DPP-4 inhibitory activity using both in vitro and in silico methods. All the compounds exhibited good DPP-4 inhibitory activity; however, their bioactivity was lower than that of sitagliptin and the lead compound. The lower bioactivity was attributed to differences in the binding interactions between the synthesized and lead compounds against the DPP-4 enzyme. Thus, the dialkyl-aminomethyl group could not effectively substitute the 3-aminopiperidine group in the lead compound.

## Data Availability

The datasets presented in this study are available upon request from the corresponding author upon submission or after publication. The data are not publicly available as they are needed for further research.
